# Visitors to Hospitals and Asylums

**Published:** 1895-05-11

**Authors:** 


### The Institutional Workshop.

### HOSPITAL ADMINISTRATION.

### VISITORS TO HOSPITALS AND ASYLUMS.

Associations for Hospital Visiting.

For many years we have persistently urged upon the public the importance of taking a deeper personal interest in the hospitals and asylums of this country. We have advocated gifts of personal service in addition to mere money gifts, and have pointed out the importance in the case of infectious hospitals, for example, of the managers throwing open these institutions when they are unoccupied, and inviting the ratepayers to visit them, so as to make them aware of the comforts and excellence of their arrangements. The late Sir Andrew Clark, in conjunction with ourselves, advocated the institution of hospital societies in connection with each congregation. These societies were to consist of members of the various churches and chapels who take an interest in the suffering and the sick. They were to co-operate with their clergymen or ministers to stimulate the congregation to larger works of benevolence on Hospital Sunday, to secure hospital accommodation for the sick of the congregation or parish, and to make an annual excursion to one of the hospitals by previous arrangement, so that they might gradually come to understand the whole system of medical relief now so admirably organised. The same idea has struck other people, but no real progress has been made with these societies, owing mainly, we believe, to a feeling on the part of the clergy and ministers of religion which, if not altered, must ultimately destroy the Hospital Sunday movement. This feeling is, that if the laity are encouraged to combine with the clergy to stimulate the interests of each congregation in one direction on behalf of the sick, or in some other direction likely to create a permanent and extended interest, then the purely religious agencies for which the clergy and ministers are responsible must suffer in cash, and consequently in credit too. We believe this view to be wholly erroneous, for as the late Canon Miller frequently pointed out, the essence of giving lies in the education of the giver. Few men or women give spontaneously to charity. The majority have to be educated up to the privilege of giving. They must learn to appreciate the height, depth, and breadth of the pleasure which the power to help others confers upon the individual when, but only when, it is exercised with discretion and kindliness. Hence we are of opinion that if the clergy and ministers of religion of all denominations, or the majority of them, grow lukewarm or callous, or even hostile in spirit to the Hospital Sunday collections, the whole of their parochial and congregational work and agencies are likely to be less vigorous and successful than ever before. The more the clergy encourage their congregations to take an interest in the hospitals and kindred benevolent societies the larger will be the amount of cash placed at their disposal for each of the many movements which they or their congregations may be interested in. We would therefore urge the clergy of all denominations to stir up their congregations to good works, and to take an individual interest in all institutions for the relief of sickness, especially in the hospitals.

A Timely Book.

One thing which we believe keeps many people from visiting the hospitals and asylums is a feeling of diffidence. They do not know how to commence, and they fear that if they attempt the work they will be unable to do any good because of their ignorance with regard to the management of these institutions. They feel, very justly, that without some knowledge and direction a visitor may be a nuisance to the officials, and anything but a comfort or help to the inmates. In these circumstance we welcome a little book entitled, "Suggestions to Hospital and Asylum Visitors," by Drs. Billings and Hurd, with an introduction by Dr. Weir-Mitchell. It is published by J. P. Lippincott Co., Philadelphia, and may be obtained at the office of The Hospital, 428, Strand, W.C. This brochure is well conceived, and is just the kind of book to do an immense amount of good in the directions indicated. It tells all who propose to give of themselves to the work of hospitals what to see in a hospital and how to see it. It ought to be as useful and profitable to members of committees, as it will undoubtedly be to the general body of governors who desire themselves to act as visitors of hospitals, either in a temporary or permanent capacity. The untrained observer is cautioned against interference in directions where his ignorance may mislead him, whilst he is shown how to gather materials for forming a just conclusion as to the relative efficiency or otherwise of each branch and portion of a hospital or asylum. It is pointed out that every hospital should contribute something to the increase and diffusion of knowledge for the benefit of other sick persons, besides those treated in its wards. It should be a means of educating and stimulating the people who contribute to its support not in medical matters, but in a knowledge of the wants of their less fortunate fellow men, and of the pleasure derivable from helping others. Visitors are warned to go to a hospital in a friendly spirit. Their first object should be to become acquainted with those charged with the immediate administration of the institution, and under their guidance, with the rules of the hospital and the methods actually used in admitting, caring for, and disposing of patients, and in obtaining and catering for the supplies. Visitors are warned that they must first understand these things and learn to look at them from the point of view of the manager and of other officials of the hospital, before they attempt to teach or to criticise. Visitors are next urged to endeavour to find out the special difficulties and troubles which these officers find most awkward to deal with, and, instead of finding fault, to acquire the habit of asking questions about doubtful points in order to call attention to matters which may have been unknown to, or overlooked by, the head of a department. Hospital visitors should ascertain whether the objects of the hospital are being attained, and whether this is being done at a reasonable cost. It is further pointed out that there are very few, if any, hospitals in which some of the medical staff do not want instruments, apparatus, drugs, or special diets which are not furnished for lack of funds. There is no hospital in which the great majority of the inmates, both employés and patients, do not desire a more varied and costly supply of food than they receive, and there is no hospital in which at times there are not demands for a greater amount of service than the employés can furnish, or than the managers think it proper to pay for. Each hospital has its own standards on these different points, and the hospital visitor must find out what that standard is, whether it is a reasonable and proper one, and whether it is being lived up to.

Subjects for Hospital Visitors.

Drs. Billings and Hurd urge that the subjects which come under the observation of hospital visitors in general will include the cleanliness and good order of the wards and the service rooms, the character of the food and mode of serving, the sufficiency of the heating and ventilation, the mode in which the work of the laundry is done, the appearance, dress, and behaviour of the nurses and other employés, the neatness of grounds and outbuildings, the completeness of the records, and the opinions of patients and their friends as to the manner in which the sick and wounded are cared for in the institution. The scrutiny of the expenditures and approval of bills may also be a part of the duty of hospital visitors. It will thus be seen that the class of visitors addressed are evidently governor visitors, as opposed to the casual caller who looks in from curiosity or by accident. It is quite manifest that many of the subjects just recited can only be usefully touched by visitors appointed at the annual meeting to make periodical visits to the institution throughout the year. It is urged that such visits should be both regular and irregular. Regular visits should be made on stated days at a certain hour, when it is to be presumed that the hospital will be seen in its best condition. Other occasional visits should be made at irregular and unexpected times, especially at times when meals are being served. It is further suggested that the visitor should sometimes make his inspection alone, and sometimes in company with the superintendent, one of the physicians, or the head nurse of a ward, being careful on the one hand, not to interfere with the work of the employés, especially in visits at irregular hours; and on the other to avoid all appearance of secret espionage. Visitors are wisely urged to see other hospitals besides the one in which they are specially interested.

Poor Law and Municipal Hospitals.

We have given these extracts from the book referred to in order to indicate the views held in America by the chief authorities as to the purpose and functions of hospital visitors. In some respects they apply with equal force to many institutions in the United Kingdom, but we are inclined to think that the most useful purpose which this book can fulfil in England at the present time is to cause the municipalities and Guardians to organise a body of visitors from amongst the ratepayers, and to take steps to throw these institutions open to their inspection. There can be no doubt that it is more difficult to obtain entrance to a Poor Law hospital, or the sick wards of the workhouse, than to any other institution in the British Empire. The consequence is that many abuses continue to thrive and increase in some of these institutions in England to-day which are opposed to some of the best interests of the public, contrary to the spirit of the age, and cruel in their oppression upon the poor inmates. These causes contribute more than anything else to the unpopularity of the English Poor Law system. Why they should be permitted to continue longer unredressed is a mystery which it is difficult to solve. Their existence benefits no one who honestly desires to promote efficiency and to make the Poor Law system a protection and comfort to the least fortunate members of the race. That the evils referred to should exist is a reflection upon our boasted civilisation, and a danger to the State. We would, therefore, commend this little book of Drs. Billings and Hurd to the careful study of the chairmen and members of the Boards of Guardians and District Councils especially, as well as to the committeemen, governors, and visitors of our asylums and hospitals. It is well calculated to do much good in many directions, and we welcome it with gratitude and appreciation.

